# Topical tranexamic acid in mastectomies on haematoma formation: prospective cohort study

**DOI:** 10.1093/bjsopen/zraf081

**Published:** 2025-07-31

**Authors:** Ali Raed Buheiri, Louise Tveskov, Laura Marie Dines, Josephine Dissing Bagge, Sören Möller, Camilla Bille

**Affiliations:** Department of Plastic Surgery, Odense University Hospital, Odense, Denmark; Department of Clinical Research, Health Sciences, University of Southern Denmark, Odense, Denmark; Department of Plastic Surgery, Odense University Hospital, Odense, Denmark; Department of Plastic Surgery, Odense University Hospital, Odense, Denmark; Department of Vascular Surgery, Rigshospitalet, Copenhagen, Denmark; OPEN Patient Data Explorative Network, Odense University Hospital and Department of Clinical Research, University of Southern Denmark, Odense, Denmark; Department of Plastic Surgery, Odense University Hospital, Odense, Denmark; Department of Clinical Research, Health Sciences, University of Southern Denmark, Odense, Denmark

## Abstract

**Background:**

Reports suggest tranexamic acid (TXA) reduces haematoma formation after breast surgery. This study investigated the effects of postoperative retrograde instillation of topical TXA through surgical drains on postoperative haematoma formation requiring surgical intervention and drain output 24 hours after mastectomy procedures.

**Methods:**

A prospective cohort study was conducted from October 2020 until September 2023, comprising two consecutive periods. In the first period, women underwent mastectomy without receiving TXA (control); in the second, women received retrograde instillation of 20 ml of 50 mg/ml TXA into the drain immediately after cavity closure. This was performed as part of a change in routine practice in April 2023. Primary endpoints collected from patient medical records were haematoma formation requiring surgical intervention, mean drain output 24 hours after the procedure, and thromboembolic events. Type of surgery, age, body mass index, smoking status, the use of blood thinners, neoadjuvant therapy, and the indication for surgery were considered patient variables that could potentially affect primary endpoints. Regression analysis was used to analyse relationships between TXA administration and haematoma occurrence and drain output, adjusting for the specified patient variables. This study was designed in accordance with STROBE guidelines.

**Results:**

Among 297 breasts (271 women) receiving topical TXA and 278 breasts (264 women) in the control group, 4 (1%) and 19 (7%) breasts, respectively, had surgical revisions due to haematoma (*P* = 0.003). Drain output within the first 24 hours after the procedure was significantly lower in the TXA than control group (mean(standard deviation) 67.6(62.4) *versus* 103.9(106.6) ml, respectively; *P* < 0.001). No thromboembolic events were reported. Women in the control group had a higher body mass index and mean age, and a higher proportion underwent axillary clearance and received neoadjuvant therapy compared with the TXA group. A higher proportion of women in the TXA group underwent sentinel node biopsy. After adjusting for these variables, significant differences remained between the two groups in haematoma rate (*P* = 0.005) and drain output (*P* = 0.001).

**Conclusion:**

Retrograde administration of 20 ml of 50 mg/ml topical TXA into the cavity after mastectomy significantly reduced the incidence of haematoma formation and drain output within the first 24 hours.

## Teaser

Topical administration of 20 ml of 50 mg/ml tranexamic acid (TXA) after mastectomy significantly reduces postoperative haematoma formation and drain output. In a study involving 297 breasts (271 women) treated with TXA and 278 breasts (264 women) that were not (control), the respective rates of surgical revision for haematoma were 1.3% and 6.8% (*P* = 0.005), and the mean drain output at 24 hours was 67.6 and 103.9 ml (*P* = 0.001). These findings highlight the effectiveness of TXA in reducing haematoma incidence and drain output during the first 24 hours after surgery.

## Introduction

Breast cancer is the most frequent cancer among women globally, accounting for one in four cancer cases^1,2^. The primary treatment option involves surgical interventions, including mastectomy or lumpectomy^3^. These procedures aim not only to treat breast cancer but also to reduce risk, thereby improving patient survival rates^4^.

In the Danish patient population, mastectomy is currently offered to approximately one-third of breast cancer patients^5^. The reported occurrence of haematomas after breast surgery varies between 1.6 and 8.1%^6–8^. Numbers are higher in patients with co-morbid conditions, who more often experience fluid accumulation, such as seroma and haematoma.

Perioperative tranexamic acid (TXA) is used across various surgical specialties and has become a standard of care to minimize bleeding in procedures associated with high blood loss^9^. TXA can be administered orally, intravenously, and, as investigated in recent studies, topically^10^. Although there are concerns regarding the potential for thromboembolic events, such as deep vein thrombosis and pulmonary embolism, with the systemic use of TXA, recent studies have shown inconsistent results, with some reporting no increased incidence^11,12^ and others suggesting a potential association^13^. The topical application of TXA in breast surgery is under investigation for its potential to mitigate bleeding and reduce haematoma formation without increasing other postoperative complications^14^. Notably, the topical use of TXA is associated with a significantly lower systemic concentration compared with intravenous administration^15^. Therefore, topical TXA would be preferred in breast surgery procedures to minimize the risk of adverse events^14,16^.

The primary aim of the present study was to investigate the impact of topical TXA on haematoma formation that requires surgical intervention after mastectomy. A secondary aim was to assess the effect of TXA on drain output within the first 24 hours (h) after surgery.

## Methods

A prospective cohort study was conducted at Odense University Hospital according to the STROBE guidelines for the reporting observational studies (*Table S1*). Following approval from the hospital’s board of directors for patient data collection, the study included all women who underwent a mastectomy, with or without axillary clearance or sentinel node biopsy between 1 October 2020 and 15 September 2023. No exclusion criteria were applied.

Data on patient demographics and possible confounding variables were collected and are presented in *Table 1*.

**Table 1:** Patient wound demographics and characteristics

	Topical TXA (*n* = 297)	No TXA (*n* = 278)
No. of patients	271	264
Age (years), mean(s.d.)	65.5(13.8)	67.2(13.6)*
BMI (kg/m^2^), mean(s.d.)	25.9(5.6)	26.7(5.3)*
Active smoker	51 (17.2%)	34 (12.2%)
Use of anticoagulants	47 (15.8%)	54 (19.4%)
**Type of procedure**		
Axillary clearance	51 (17.2%)	81 (29.1%)*
Sentinel node	206 (69.4%)	167 (60.1%)*
Mastectomy alone	40 (13.5%)	30 (10.8%)
Neoadjuvant therapy	56 (18.9%)	74 (26.6%)*
**Indication for mastectomy**		
Prophylactic, *n*	9 (3.0%)	6 (2.2%)
Curative, *n*	288 (97.0%)	272 (97.8%)

Values are *n* (%) unless otherwise indicated. Data for wound demographics and characteristics are based on the number of breasts in each of the topical TXA and control groups, not the number of patients. TXA, tranexamic acid; SD, standard deviation; BMI, body mass index. *P  *<* 0.05 (independent *t* tests for continues variables; χ^2^ tests for categorical variables).

Patients treated between 1 October 2020 and 1 April 2022, did not receive any TXA and served as the control group. After a change in routine clinical practice at Odense University Hospital starting 1 April 2022, all mastectomy patients were to receive topical administration of 10 ml of 1 g TXA diluted in 10 ml saline solution (NaCl) upon wound closure. Subsequently, women received retrograde instillation of 20 ml of 50 mg/ml TXA into the drain for each breast. The drains were clamped for 30 min before being opened into the drainage bag. Patients who underwent a mastectomy and received this treatment between 1 April 2022 and 15 September 2023 were included in this study as the TXA group.

In all patients undergoing mastectomy, a surgical drain was inserted into the mastectomy pocket. This drain remained in place until the collected fluid turned serous or the daily output was less than 75 ml. The incidence of haematoma requiring surgical intervention under general anaesthesia up until discharge and drain output within the first 24 h after the surgery were analysed in both groups.

The surgical team, comprising breast surgeons, was unchanged throughout the 3-year study period.

### Statistical analysis

Descriptive statistics were used to explore patient wound demographics and clinical characteristics. Mean, standard deviation (s.d.), and percentage values were calculated to provide a comprehensive overview, encompassing procedure types (mastectomy, with or without axillary clearance or sentinel node biopsy), indications for surgery (curative or prophylactic), and the number of breasts, because the calculations are based on the number of operated breasts, but some patients had bilateral procedures. The significance of differences between groups was calculated using *t* tests for continues variables and χ^2^ tests for categorical variables. Relationships between TXA administration and outcomes were evaluated using Fisher’s exact test and χ^2^ tests for haematoma and independent *t* tests for drain output. A *P*-value < 0.05 was considered statistically significant.

Regression analysis was conducted to assess the impact of TXA on haematoma formation and drainage output adjusted for variables that may affect both haematoma formation and drain volume. A logistic regression model was used to assess predictors of haematoma presence, and a linear regression model was used to investigate predictors of drain output. Each regression analysis was performed after calculation of descriptive statistics and preliminary univariate analysis. This analysis adjusted for potential confounders, including age, body mass index (BMI), smoking status, the use of anticoagulants, adjuvant therapy, and type of procedure. Subgroup analyses were stratified by operation type to assess the differential effects of TXA.

Data were stored and managed on secure servers of Statistics Denmark, using Stata ® / SE 18 (StataCorp, College Station, TX, USA) for analysis. Data were only extracted after anonymization.

## Results

**Fig. 1:**
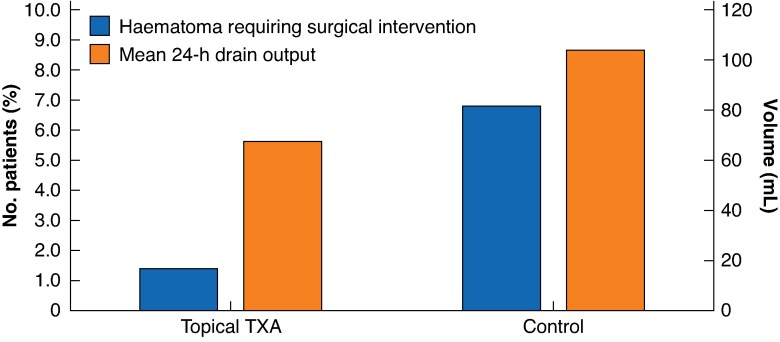
Diagram of haematoma formation and 24 hours mean drain output in each group TXA, tranexamic acid.

Patient wound characteristics are presented in *Table 1*; an overview of each procedure and indication is provided in *Table S2*. In all, 575 breasts (535 patients) underwent mastectomy between 1 October 2020 and 15 September 2023; of these, 297 breasts (271 patients) were in the group that received topical TXA and 278 breasts (274 patients) were in the control group (*Fig. 1*).

Four (1.3%) surgical revisions due to haematoma were managed in the TXA group, compared with 19 (6.8%) in the control group (odds ratio (OR) 0.19; *P* = 0.003). The mean drain output within the first 24 h after the mastectomy was 67.6 ml in the TXA group and 103.9 ml in the control group (mean difference 36.3 ml; 95% confidence interval (c.i.) −51.46 to −21.22  ml; *P* < 0.001; *Table 2*), representing a 34.9% reduction in drain output in the TXA group. No thromboembolic events were registered in either group.

**Table 2:** Postoperative complications

	Topical TXA (*n* = 297)	Control (*n* = 278)	Topical TXA *versus* control			
			Effect*	*P*	Adjusted difference†	
					Effect*	*P*
Haematoma	4 (1.3%)	19 (6.8%)	OR 0.19 (0.06, 0.56)	0.003	OR 0.20 (0.07, 0.62)	0.005
Drain output after 24 h (ml), mean(s.d.)	67.6(62.4)	103.9(106.6)	Diff −36.34 (−51.46, −21.22)	<0.001	Diff −25.6 (−40.77, −10.42)	<0.001
Thromboembolic events	0 (0%)	0 (0%)				

Values are *n* (%) unless otherwise indicated. Data for postoperative complications are based on the number of breasts in each of the topical TXA and control groups. *Values in parentheses are 95% confidence intervals. †Regression analysis model adjusted for age, body mass index, smoking, blood thinning, and neoadjuvant chemotherapy, procedure, and indication. TXA, tranexamic acid; OR, odds ratio; s.d., standard deviation; Diff, difference.

Because several variables differed significantly between groups (*Table 1*), regression analyses were performed to adjust for these variables. After adjusting for confounding variables using logistic regression, topical TXA was found to significantly reduce haematoma formation (OR 0.20; *P* = 0.005) and linear regression showed a significant reduction in 24-h drain output in all breasts in the TXA group compared with the control group (mean difference −25.6 ml; *P* = 0.001; *Tables S3*, *S4*).

Regression analysis revealed that the use of anticoagulants increased the risk of haematoma formation (OR 3.23; *P*  *=* 0.013). Moreover, each additional year of age and each 1-unit increase in BMI were associated with higher drain output (difference 0.82 ml (*P* < 0.001) and 3.79 ml (*P* < 0.001), respectively); conversely, smoking was associated with a lower drain output within the first 24 h (difference −15.01; *P* < 0.044). Axillary clearance was associated with a significantly higher drain output compared with other procedures (difference 39.77 ml; *P* = 0.001; *Tables S3*, *S4*).

## Discussion

The off-label use of TXA in surgical procedures has gained increasing recognition for its potential to reduce bleeding. The findings of the present study align with previous randomized clinical trials^14,17^ that have demonstrated the significant efficacy of topical TXA in reducing drain output and suggested a reduction in haematoma formation. Building upon these findings, the present study is the largest to date on the use of TXA in patients undergoing mastectomy. After adjusting for patient variables that may affect both haematoma rates and drain production, this study demonstrated that the topical application of TXA significantly reduces the incidence of haematoma requiring surgical intervention as well as the amount of drainage fluid in mastectomy patients compared with the control group. This sets the stage for future research assessing the most optimal route of administration and dosage of TXA.

Regression analyses revealed that the use of anticoagulants was associated with an increased incidence of haematoma formation in the present study. In addition, the type of surgical procedure, particularly mastectomy with axillary clearance, and higher BMI, older age, and smoking status showed significant associations with drain output.

Current treatment guidelines do not specify the uses for, dosage, and administration method of topical TXA. This lack of standardization has led to various practices in clinical settings. The method of TXA administration in the present study aligns with methods documented in other breast surgery research^18,19^, yet differs from other studies that either moistened the wound before closure or soaked the breast pocket before implant placement^14,17,20^. In contrast, the approach used in the present study involved retrograde TXA administration through the drain after wound closure, minimizing wound exposure and reducing surgery time. Despite direct application providing precise TXA placement, the retrograde approach through the drain used in the present study offers more consistent medication delivery, similar to rinsing the breast pocket^21^.

The concentrations of TXA used also vary between studies. In the study of Yao *et al*.^20^, patients received 20 ml of 50 mg/ml TXA, whereas other studies have reported administering 20 ml of 25 mg/ml TXA^14,16^, 30 mg/ml TXA^17^, and 110 ml of 9.09 mg/ml TXA^22^. These studies showed statistically significant reductions in drain output and varying degrees of reductions in haematoma formation compared with control groups. This contributes to a broader understanding of effective dosages of TXA and application methods. Furthermore, clinical studies^15,23^ provide evidence supporting the safety of TXA concentrations below 100 mg/ml for topical applications. No thromboembolic events or other major complications were registered in either group in the present study.

TXA is well documented and widely used across various medical specialties to reduce bleeding. It can be administrated orally, intravenously, and topically in different settings. However, topical administration of TXA has gained interest due to its higher local effect while reducing systemic side effects. The primary mechanism of action of TXA is its antifibrinolytic effect, which reduces bleeding by inhibiting the activation of plasminogen to plasmin, thereby preventing the breakdown of fibrin clots. This mechanism explains the observed reduction in postoperative bleeding and drain output, because TXA stabilizes clots and reduces microhaemorrhages^24^. Nevertheless, it is crucial to acknowledge that the efficacy of pharmacological interventions, such as TXA, cannot outweigh the importance of surgical skills and techniques. The findings of the present study show that topical TXA significantly reduced haematoma formation and drain output in patients who had undergone mastectomy. However, the integration of TXA into mastectomy procedures at the Department of Plastic Surgery, Odense University Hospital, Denmark, may have unconsciously enhanced surgical precision, focusing on achieving proper haemostasis; it could have contributed to the differences observed between the two groups. Conversely hand, no surgeon ever closes if there is ongoing bleeding from the cavity.

Given the nature of the present study, it is important to recognize its limitations in isolating the specific effects of TXA.

The study has inherent weaknesses compared with a randomized clinical trial, particularly in terms of validity. The same surgeons performed the procedures throughout the study, executing them in the exact manner they have for years. They used the same instruments and adhered to the same postoperative regimen. This consistent environment helps minimize variables that could typically compromise the study's validity, ensuring that the results are as reliable as possible under the circumstances.

Despite these limitations, this research offers valuable insights into the benefits of topical TXA use, setting the stage for further studies to identify the most effective dosages and administration methods. Thus, topical TXA emerges as a valuable tool in surgical management.

## Supplementary Material

zraf081_Supplementary_Data

## Data Availability

The data underlying this study are not publicly available due to ethical restrictions protecting patient confidentiality.
