# Supplementary material for: Topical tranexamic acid in mastectomies on haematoma formation: prospective cohort study
Source: BJS Open. 2025 Jul 31;9(4):zraf081. doi: 10.1093/bjsopen/zraf081 (PMC12312098; doi:10.1093/bjsopen/zraf081)
Supplement: zraf081_Supplementary_Data [file zraf081_supplementary_data.docx]

**Topical Tranexamic acid in mastectomies on hematoma formation: Prospective cohort study**

Ali Raed Buheiri^1,2^, Louise Tveskov^1^, Laura Marie Dines^1^, Josephine Dissing Bagge^3^, Sören Möller^4^, Camilla Bille^1,2^

^1^Department of Plastic Surgery, Odense University Hospital, Odense, Denmark

^2^University of Southern Denmark, Odense, Denmark

^3^Department of Vascular Surgery, Rigshospitalet, Copenhagen, Denmark

^4^OPEN Patient Data Explorative Network, Odense University Hospital and Department of Clinical Research, University of Southern Denmark, Odense, Denmark

Corresponding author

Ali Raed Buheiri

E-mail: ali.raed.buheiri@rsyd.dk

Address: Department of Plastic Surgery, Odense University Hospital and Lillebaelt Hospital, 5000, Odense, Denmark.

**Supplementary Materials - Index**

| **Supplementary Figures and Tables** |  |
| --- | --- |
| Table S1 | *pag. 2-3* |
| Table S2 | *pag. 4* |
| Table S3  Table S4 | *pag. 5*  *pag. 6* |

**Supplementary Figures and Tables**

**Supplementary material**

Table S1. STROBE Statement - Checklist of items that should be included in reports of cohort studies.

|  | | Item No | Recommendation | Page No |
| --- | --- | --- | --- | --- |
| **Title and abstract** | | 1 | (*a*) Indicate the study’s design with a commonly used term in the title or the abstract |  |
|  |  |  | (*b*) Provide in the abstract an informative and balanced summary of what was done and what was found |  |
| Introduction | | | | |
| Background/rationale | | 2 | Explain the scientific background and rationale for the investigation being reported |  |
| Objectives | | 3 | State specific objectives, including any prespecified hypotheses |  |
| Methods | | | | |
| Study design | | 4 | Present key elements of study design early in the paper |  |
| Setting | | 5 | Describe the setting, locations, and relevant dates, including periods of recruitment, exposure, follow-up, and data collection |  |
| Participants | | 6 | (*a*) Give the eligibility criteria, and the sources and methods of selection of participants. Describe methods of follow-up |  |
|  |  |  | (*b*) For matched studies, give matching criteria and number of exposed and unexposed |  |
| Variables | | 7 | Clearly define all outcomes, exposures, predictors, potential confounders, and effect modifiers. Give diagnostic criteria, if applicable |  |
| Data sources/ measurement | | 8* | For each variable of interest, give sources of data and details of methods of assessment (measurement). Describe comparability of assessment methods if there is more than one group |  |
| Bias | | 9 | Describe any efforts to address potential sources of bias |  |
| Study size | | 10 | Explain how the study size was arrived at |  |
| Quantitative variables | | 11 | Explain how quantitative variables were handled in the analyses. If applicable, describe which groupings were chosen and why |  |
| Statistical methods | | 12 | (*a*) Describe all statistical methods, including those used to control for confounding |  |
|  |  |  | (*b*) Describe any methods used to examine subgroups and interactions |  |
|  |  |  | (*c*) Explain how missing data were addressed |  |
|  |  |  | (*d*) If applicable, explain how loss to follow-up was addressed |  |
|  |  |  | (*e*) Describe any sensitivity analyses |  |
| Results | | | |  |
| Participants | | 13* | (a) Report numbers of individuals at each stage of study—eg numbers potentially eligible, examined for eligibility, confirmed eligible, included in the study, completing follow-up, and analysed |  |
|  |  |  | (b) Give reasons for non-participation at each stage |  |
|  |  |  | (c) Consider use of a flow diagram |  |
| Descriptive data | | 14* | (a) Give characteristics of study participants (eg demographic, clinical, social) and information on exposures and potential confounders |  |
|  |  |  | (b) Indicate number of participants with missing data for each variable of interest |  |
|  |  |  | (c) Summarise follow-up time (eg, average and total amount) |  |
| Outcome data | | 15* | Report numbers of outcome events or summary measures over time |  |
| Main results | 16 | (*a*) Give unadjusted estimates and, if applicable, confounder-adjusted estimates and their precision (eg, 95% confidence interval). Make clear which confounders were adjusted for and why they were included | |  |
|  |  | (*b*) Report category boundaries when continuous variables were categorized | |  |
|  |  | (*c*) If relevant, consider translating estimates of relative risk into absolute risk for a meaningful time period | |  |
| Other analyses | 17 | Report other analyses done—eg analyses of subgroups and interactions, and sensitivity analyses | |  |
| Discussion | | | | |
| Key results | 18 | Summarise key results with reference to study objectives | |  |
| Limitations | 19 | Discuss limitations of the study, taking into account sources of potential bias or imprecision. Discuss both direction and magnitude of any potential bias | |  |
| Interpretation | 20 | Give a cautious overall interpretation of results considering objectives, limitations, multiplicity of analyses, results from similar studies, and other relevant evidence | |  |
| Generalisability | 21 | Discuss the generalisability (external validity) of the study results | |  |
| Other information | | | | |
| Funding | 22 | Give the source of funding and the role of the funders for the present study and, if applicable, for the original study on which the present article is based | |  |

*Give information separately for exposed and unexposed groups.

Table S2. Patient characteristics and results of each procedure and indication.

|  | All breasts | | | Mastectomy | | | Mastectomy + SN | | | Mastectomy + Axillary clearance | | |
| --- | --- | --- | --- | --- | --- | --- | --- | --- | --- | --- | --- | --- |
| Breasts | **TXA = 297** | | **Control = 278** | **TXA = 40** | | **Control = 30** | **TXA = 206** | **Control = 167** | | **TXA = 51** | **Control = 81** | |
| Age | 65.5 $\pm$13.8 | | 67.2 $\pm$ 13.6 | 58.4 $\pm$ 17.6 | | 64.5 $\pm$ 13.8 | 67.5 $\pm$ 12.3 | 68.8 $\pm$ 12.7 | | 62.5 $\pm$ 14.2 | 64.9 $\pm$ 15.0 | |
| BMI | 25.9 $\pm$ 5.6 | | 26.7 $\pm$ 5.3 | 25.4 $\pm$ 5.3 | | 26.9 $\pm$ 5.2 | 25.8 $\pm$ 5.6 | 26.0 $\pm$ 5.0 | | 27.1 $\pm$ 5.6 | 28.1 $\pm$ 5.7 | |
| Active smoker | 51 (17%) | | 34 (12%) | 6 (15%) | | 4 (13%) | 35 (17%) | 23 (14%) | | 10 (20%) | 7 (9%) | |
| Use of anticoagulants | 47 (16%) | | 54 (19%) | 5 (13%) | | 5 (17%) | 33 (16%) | 31 (19%) | | 9 (18%) | 18 (22%) | |
| Neoadjuvant therapy | 56 (19%) | | 74 (27%) | 5 (13%) | | 3 (10%) | 35 (17%) | 32 (19%) | | 16 (31%) | 39 (48%) | |
| Indication: |  |  |  |  |  |  |  |  |  |  |  |  |
| Prophylactic |  | | | 7 | | 4 | 2 | 2 | |  |  | |
| Curative |  |  |  | 33 | | 26 | 204 | 165 | | 51 | 81 | |
| Outcomes: |  |  |  |  |  |  |  |  |  |  |  |  |
| Hematoma | 4 (1%) | | 19 (7%) | 2 (5%) | | 2 (7%) | 2 (1%) | 12 (7%) | | 0 (0%) | 5 (6%) | |
| Odds ratio, (*p)* | 0.19 (p = 0.003) | | | 0.74 (p = 0.766) | | | 0.13 (p = 0.007) | | | 1 | | |
| Drain output after 24 hours, ml | 67.6 ml | | 103.9 ml | 58 ml | | 85.5 ml | 60.7 ml | 90.3 ml | | 103.0 ml | 138.9 ml | |
| Mean difference, (*p*) | -36.3 ml (p < 0.001) | | | -27.5 ml (p = 0.263) | | | -29.6 ml (p = 0.003) | | | -35.8 ml (p = 0.014) | | |
| Thromboembolic events | 0 (0%) | 0 (0%) | | 0 (0%) | 0 (0%) | | 0 (0%) | | 0 (0%) | 0 (0%) | | 0 (0%) |

TXA = tranexamic acid, BMI = body mass index, M = mastectomy, SN = sentinel node biopsy, Str. err. = Standard error, CI = Confidence interval

Table S3. Logistic regression model on hematoma formation stratified on surgical

procedure.

| Hematoma formation requiring surgical intervention stratified on procedure | | | | | | | | | | |
| --- | --- | --- | --- | --- | --- | --- | --- | --- | --- | --- |
|  | **All breasts** | | | | | **Mastectomy** | | | | |
| Variable | **Odds ratio** | **Std. err.** | **z** | **P>\|z\|** | **[95% CI. Interval]** | **Odds ratio** | **Std. err.** | **z** | **P>\|z\|** | **[95% CI. Interval]** |
| Topical TXA | 0.20 | 0.115 | -2.81 | 0.005 | [0.07, 0.62] | 1.03 | 1.056 | 0.03 | 0.979 | [0.14, 7.70] |
| Age | 1.01 | 0.019 | 0.54 | 0.590 | [0.97, 1.05] | 1.07 | 0.034 | 2.22 | 0.026 | [1.01, 1.14] |
| BMI | 1.02 | 0.029 | 0.63 | 0.526 | [0.96, 1.08] | 1.18 | 0.067 | 2.81 | 0.005 | [1.05, 1.31] |
| Active smoker | 0.64 | 0.479 | -0.59 | 0.552 | [0.15, 2.77] | 1 |  |  |  |  |
| Use of anticoagulants | 3.23 | 1.522 | 2.49 | 0.013 | [1.28, 8.14] | 1.62 | 2.143 | 0.36 | 0.716 | [0.12, 21.69] |
| Neoadjuvant therapy | 1.19 | 0.683 | 0.29 | 0.769 | [0.38, 3.66] | 1 |  |  |  |  |
| Procedure: |  |  |  |  |  |  |  |  |  |  |
| m |  |  |  |  |  | 1 |  |  |  |  |
| m + sn | 0.35 | 0.257 | -1.43 | 0.152 | [0.08, 1.47] |  |  |  |  |  |
| m + axillary clearance | 0.45 | 0.290 | -1.24 | 0.214 | [0.12, 1.60] |  |  |  |  |  |
| _cons | 0.04 | 0.049 | -2.40 | 0.017 | [<0.01, 0.54] | 8.70e-06 | 2.7e-05 | -3.82 | 0.0001 | [2.21e-08,  <0.01] |
|  | **Mastectomy + SN** | | | | | **Mastectomy + Axillary clearance** | | | | |
| Variable | **Odds ratio** | **Std. err.** | **z** | **P>\|z\|** | **[95% CI. Interval]** | **Odds ratio** | **Std. err.** | **z** | **P>\|z\|** | **[95% CI. Interval]** |
| Topical TXA | 0.14 | 0. 109 | -2.46 | 0.014 | [0.03, 0.67] | 1 |  |  |  |  |
| Age | 0.99 | 0.024 | 0.58 | 0.563 | [0.94, 1.03] | 1.01 | 0.036 | 0.37 | 0.715 | [0.95, 1.09] |
| BMI | 1.01 | 0.042 | 0.24 | 0.810 | [0.93, 1.10] | 0.98 | 0.078 | -0.30 | 0.765 | [0.84, 1.14] |
| Active smoker | 0.89 | 0.713 | -0.15 | 0.884 | [0.19,  4.28] | 1 |  |  |  |  |
| Use of anticoagulants | 4.59 | 2.709 | 2.58 | 0.010 | [1.44, 14.60] | 2.53 | 2.264 | 1.04 | 0.299 | [0.44, 14.62] |
| Neoadjuvant therapy | 0.72 | 0.717 | -0.33 | 0.743 | [0.10, 5.05] | 2.04 | 1.832 | 0.80 | 0.426 | [0.35, 11.85] |
| Procedure: |  |  |  |  |  |  |  | | | |
| m |  |  |  |  |  |  |  |  |  |  |
| m + sn | 1 |  |  |  |  |  |  |  |  |  |
| m + axillary clearance |  |  |  |  |  | 1 |  |  |  |  |
| _cons | 0.10 | 0.20 | -1.15 | 0.25 | [<0.01, 4.95] | 0.03 | 0.0814 | -1.36 | 0.172 | [<0.01, 4.46] |

TXA = tranexamic acid, BMI = body mass index, M = mastectomy, SN = sentinel node biopsy, Str. err. = Standard error, CI = Confidence interval

Table S4. Linear regression model drain output stratified on surgical procedure.

| Drain output stratified on procedure | | | | | | | | | | |
| --- | --- | --- | --- | --- | --- | --- | --- | --- | --- | --- |
|  | **All breasts** | | | | | **Mastectomy** | | | | |
| Variable | **Coefficient** | **Std. err.** | **t** | **P>\|t\|** | **[95% CI. Interval]** | **Coefficient** | **Std. err.** | **t** | **P>\|t\|** | **[95% CI. Interval]** |
| Topical TXA | -25.59 | 7. 72 | -3.31 | 0.001 | [-40.77, -10.42] | -7.48 | 18.76 | -0.40 | 0.692 | [-44.97, 30.02] |
| Age | 0.82 | 0.22 | 3.76 | <0.001 | [0.39, 1.25] | 0.79 | 0.43 | 1.87 | 0.067 | [-0.06, 1.66] |
| BMI | 3.79 | 0.67 | 5.68 | <0.001 | [2.48, 5.11] | 6.54 | 1.37 | 4.78 | <0.001 | [3.80, 9.27] |
| Active smoker | -15.01 | 7.43 | -2.02 | 0.044 | [-29.60, -0.42] | 32.81 | 14.55 | 2.26 | 0.028 | [3.74, 61.88] |
| Use of anticoagulants | 22.23 | 12.21 | 1.82 | 0.069 | [-1.76, 46.22] | 72.91 | 49.56 | 1.47 | 0.146 | [-26.12, 171.95] |
| Neoadjuvant therapy | -0.20 | 7.81 | -0.03 | 0.979 | [-15.54, 15.13] | 12.15 | 16.70 | 0.73 | 0.470 | [-21.23, 45.53] |
| Procedure: |  |  |  |  |  |  |  |  |  |  |
| m |  |  |  |  |  | 0 |  |  |  |  |
| m + axillary clearance | 39.77 | 11.88 | 3. 35 | 0.001 | [16.44, 63.11] |  |  |  |  |  |
| m + sn | -1.63 | 10.11 | -0.16 | 0.872 | [-21.49, 18.23] |  |  |  |  |  |
| _cons | -65.26 | 21.78 | -3.00 | 0.003 | [-108.10, -22.44] | -160.19 | 42.69 | -3.75 | <0.001 | [ -245.51, -74.88] |
|  | **Mastectomy + SN** | | | | | **Mastectomy + Axillary clearance** | | | | |
| Variable | **Coefficient** | **Std. err.** | **t** | **P>\|t\|** | **[95% CI]** | **Coefficient** | **Std. err.** | **t** | **P>\|t\|** | **[95% CI]** |
| Topical TXA | -27.96 | 9.88 | -2.83 | 0.005 | [-47.39, -8.54] | -24.74 | 14.16 | -1.75 | 0.083 | [-52.77, 3.29] |
| Age | 0.88 | 0.298 | 2.94 | 0.004 | [0.29, 1.46] | 0.85 | 0.47 | 1.82 | 0.072 | [-0.08, 1.78] |
| BMI | 3.05 | 0.81 | 3.75 | <0.001 | [1.45, 4.65] | 4.26 | 0.95 | 4.51 | <0.001 | [2.39, 6.13] |
| Active smoker | -16.12 | 9.35 | -1.72 | 0.086 | [-34.51, 2.27] | -35.09 | 14.39 | -2.44 | 0.016 | [-63.58, -6.61] |
| Use of anticoagulants | 16.09 | 15.49 | 1.04 | 0.300 | [-14.38, 46.56] | 15.66 | 19.52 | 0.80 | 0.424 | [-22.97, 54.29] |
| Neoadjuvant therapy | -1.17 | 9.22 | -0.13 | 0.899 | [-19.31, 16.96] | 0.35 | 15.47 | 0.02 | 0.982 | [-30.26, 30.96] |
| Procedure: |  |  | | | |  |  |  |  |  |
| m |  |  |  |  |  |  |  |  |  |  |
| m + sn | 0 |  |  |  |  |  |  |  |  |  |
| m + axillary clearance |  |  |  |  |  | 0 |  | | | |
| _cons | 117.6 | 30.83 | -1.68 | 0.095 | [-105.04, 8.39] | -37.21 | 41.596 | -0.89 | 0.373 | [-119.53, 45.10] |

TXA = tranexamic acid, BMI = body mass index, M = mastectomy, SN = sentinel node biopsy, Str. err. = Standard error, CI = Confidence interval

|  |  |
| --- | --- |
|  |  |
